# Liquid- and Semisolid-Filled Hard Gelatin Capsules Containing Alpha-Lipoic Acid as a Suitable Dosage Form for Compounding Medicines and Dietary Supplements

**DOI:** 10.3390/pharmaceutics16070892

**Published:** 2024-07-04

**Authors:** Jelena Jovičić-Bata, Nemanja Todorović, Veljko Krstonošić, Ivan Ristić, Zorana Kovačević, Milana Vuković, Mladena Lalić-Popović

**Affiliations:** 1 Department of Pharmacy, Faculty of Medicine Novi Sad, University of Novi Sad, 21000 Novi Sad, Serbia; jelena.jovicic-bata@mf.uns.ac.rs (J.J.-B.); veljko.krstonosic@mf.uns.ac.rs (V.K.); milana.vukovic@mf.uns.ac.rs (M.V.); mladena.lalic-popovic@mf.uns.ac.rs (M.L.-P.); 2 Faculty of Technology Novi Sad, University of Novi Sad, 21000 Novi Sad, Serbia; ivan.ristic@uns.ac.rs; 3 Department of Veterinary Medicine, Faculty of Agriculture, University of Novi Sad, 21000 Novi Sad, Serbia; zorana.kovacevic@polj.edu.rs; 4 Centre for Medical and Pharmaceutical Investigations and Quality Control (CEMPhIC), Faculty of Medicine Novi Sad, University of Novi Sad, 21000 Novi Sad, Serbia

**Keywords:** thioctic acid, dissolution test, rheology, FTIR, colloidal silicon dioxide, polyethylene glycol (PEG-400), extemporaneous formulations, dosage forms and excipients, compounding technologies

## Abstract

Liquid-filled hard gelatin capsules may have pertinent advantages both for therapeutic effect and extemporaneous preparations of medicines. Alpha lipoic acid is a substance used in medicines and dietary supplements and there is a need for creating an appropriate formulation which would be suitable for each individual patient or consumer. Based on its biopharmaceutical and physical chemical characteristics, eight different capsule formulations were designed and characterized. Silicon dioxide was added to form a semisolid content and prevent leakage. The formulation filled with alpha lipoic acid solution in polyethylene glycol 400 showed the best performance. Although the addition of silicon dioxide to the formulation with polyethylene glycol 400 led to a change in both flow character and viscosity, the release rate did not show a statistically significant decrease (more than 85% of content was released after 5 min testing). Applied technique is a simple and an appropriate approach for compounding and could be used for other substances with similar properties.

## 1. Introduction

**Figure 1:**
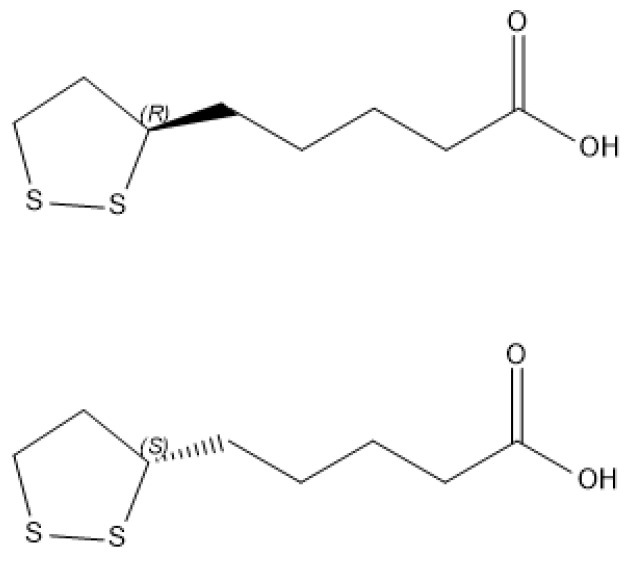
Chemical structure of alpha-lipoic acid (ALA); the levorotatory isomer S-(−)-ALA and the dextrorotatory isomer R-(+)-ALA.

Alpha-lipoic acid (ALA, thioctic acid) is a sulfur-containing organic acid, produced in the mitochondria of plant and animal species from octanoic acid and cysteine as a source of sulfur. Due to its amphiphilic properties, ALA is ubiquitous, present in cell membranes and cytosol [1]. There are indications that Western diets do not provide ample amounts of ALA [2]. The endogenous synthesis of ALA regulated by lipoic acid synthase in humans is possibly insufficient [3], dependent on lipoic acid synthase activity [4] and decreases with age [3,5]. Exogenous sources of ALA are therefore required. The naturally occurring, biologically active form of ALA is its R-enantiomer, but commercial preparations of ALA often contain a mixture of R and S-enantiomers (Figure 1). S-enantiomer is a byproduct of ALA industrial synthesis with no known negative health effects [6,7]. R-enantiomer might be slightly better absorbed and cleared from the body slower than the S-form [2]. Additionally, isolated R-form is more expensive.

In the human body, the fraction of ALA covalently bound to proteins is an important part of several mitochondrial enzyme complexes engaged in energy production [8,9]. Unbound ALA acts as a “universal antioxidant” [6], active inside and outside of cells, both in its oxidized and reduced (dihydrolipoic acid/DHLA) forms [10]. ALA scavenges reactive oxygen species (ROS), regenerates other antioxidants such as glutathione, vitamin C and vitamin E, chelates selected metal ions, affects the activity of several redox enzyme systems and possibly modulates some signaling pathways including the insulin pathway [11,12]. ALA has also been shown to display anti-inflammatory actions by suppressing the production of pro-inflammatory proteins [13,14,15,16,17,18].

ALA is available to consumers in various oral dosage forms, e.g., capsules, both soft and hard, immediate release tablets, film-coated gastroresistant tablets [19,20], and is registered both as medicines and dietary supplements (DS). These products differ significantly. In most countries, DS are regulated as food and are freely available for purchase. Strict conforming to pharmacopeial standards in terms of quality testing enforced on medicines by pharmacopeias (e.g., dissolution testing, disintegration testing, and friability) are usually not matched by DS regulations. DS can be registered and marketed without quality testing and their efficacy being established.

Doses of ALA in DS range from 10 mg to 600 mg, the latter being a dose equal to the one used in medicines. Daily doses of ALA used in treatment of diabetes neuropathy are usually 300 to 1800 mg, but the optimal doses for other potential “indications” have not been established. Clinical studies of ALA assessed the use of 100 to 2400 mg of ALA per day in different disorders [21]. Furthermore, there are indications that the dosing of ALA should be adjusted in patients with severe renal function impairment [22]. It should be noted that ALA can trigger insulin autoimmune syndrome (IAS). Namely, a sulfhydryl compound like ALA can help dissociate S-S bonds within insulin molecules and expose specific insulins’ amino acid sequence (Ile-Leu-Gln), which acts as an antigen, thereby provoking an allergic reaction [23]. The development of IAS is strongly connected to sulfhydryl with specific genotypes in the blood type marker Human Leucocyte Antigen (HLA). Furthermore, Gullo et al. estimated that increased use of ALA in food supplements led to an increased frequency of IAS in Italy [24].

ALA is a weak acid with a pKa value between 4.76 and 5.4 [25]. As a lipophilic compound, it has a logP value of 2.11 [26]. ALA preparations should be taken on an empty stomach, 30 min before or 2 h after a meal, as food has been shown to impair its absorption [27]. Due to ALA properties, formulation of its dosage forms presents a challenge for the pharmaceutical industry. Namely, it is poorly soluble in water, unstable in the presence of gastric acid, and extensively metabolized in the liver, which results in its bioavailability of only about 30% [3]. It is classified as a class II substance in the biopharmaceutics classification system (BCS) [20,28]. The bioavailability of ALA is better in the presence of substances that increase its solubility and in the form of liquid formulations [29,30]. Hence, the formulation characteristics of its dosage forms can significantly affect its bioavailability and, consequently, its effects.

Taking into account the general risk of IAS and data from animal studies, the calculated upper safe dose per person per day is 42 mg [31]. Although higher doses have not been linked to serious adverse effects, the European Food Safety Authority suggests that 150–200 mg of ALA per day causes safety concerns [32,33,34]. Though there is plethora of oral dosage forms with ALA on the market worldwide, there is a need for a specific strength or dosage that might not be commercially available. Also, the removal of some ingredients that patients are allergic to might be required.

However, ALA is chemically unstable under light and heat. The decomposition of ALA is accompanied by an unpleasant odor due to the sulfur it contains. At temperatures that are greater than its melting point (59–62 °C), immediate polymerization occurs [35]. Thus, the US Food and Drug Administration categorized ALA into category 1 in section 305B of the FD&C Act, which are bulk drug substances that have physical and chemical properties that could have an impact on the performance of the compounded product. ALA is also under consideration to be allowed for use in compounding by 503A facilities [36].

Thus, the aim of this work was the preparation of custom-made simple dosage forms with ALA and their complete characterization. Compounding was performed using hard gelatin capsules that are suitable in pharmaceutical practice. Two formulations contained solid powder filling, three formulations were liquid, and three were compounded as semisolid formulations.

## 2. Materials and Methods

### 2.1. Materials

The following chemicals were used in the experiment: USP-grade alpha-lipoic acid (ALA, Farmalabor, Canosa di Puglia, Italy), CapsuLac^®^ 60 (Meggle, Wasserburg am Inn, Germany), Excipient II (Farmalabor, Canosa di Puglia, Italy; composition: pregelatinized corn starch, magnesium stearate, micronized silicon dioxide, micronized talc), absolute ethanol (Zorka Pharma-Hemija, Šabac, Serbia), polyethylene glycol 400 (PEG 400, Alfa Aesar, Kandel, Germany), caprylic/capric triglycerides (Comcen, Belgrade, Serbia), colloidal silicon dioxide (Centrohem, Stara Pazova, Serbia), hard gelatin capsules size 0 (Farmalabor, Canosa di Puglia, Italy; composition: indigo FD&C Blue 2 (E132), titanium dioxide, and yellow iron oxide (E172), gelatin). Methanol (Lachner, Neratovice, Czech Republic) was used for the content determination test. The dissolution rate was tested in a 0.5% sodium lauryl sulfate (SLS, Centrohem, Stara Pazova, Serbia) water solution. Purified water used in all experiments was obtained by distillation (AC-L8, Optic Ivymen System, J.P. Selecta, Barcelona, Spain) at the Department of Pharmacy, Faculty of Medicine Novi Sad.

### 2.2. Capsules Formulation

Capsules were compounded according to the standard operating procedure of the Pharmaceutical Technology Laboratory at the Department of Pharmacy, Faculty of Medicine Novi Sad. A manual capsule-filling machine (Farmalabor, Canosa di Puglia, Italy) with a size 0 capsule kit (V = 0.68 mL) was used. The loading capacities were determined for the powder components after standard filling with pure substances (*n* = 20) and they were 261.70 ± 29.43 mg, 479.07 ± 6.75 mg, and 443.29 ± 13.70 mg for ALA, CapsuLac^®^ 60, and Excipient II, respectively.

Three solid-filled capsule formulations were compounded for comparation with new liquid- and semisolid-filled capsules. Formulation F_0_ contained only 100 mg of ALA. Formulations F_1_ and F_2_ contained CapsuLac^®^ 60 and Excipient II as fillers and 100 mg of ALA. Masses of fillers were calculated using Equation (1):

m = C − D,(1)

where m is the mass of the filler needed to compound one capsule, C is the experimentally determined loading capacity of the filler, and D is the dose of ALA needed to compound one capsule (100 mg).

The masses of pure substances required for the compounding of 50 capsules were sifted through a test sieve 355 µm on a sieve shaker (AS 200 control, Retsch, Haan, Germany) and weighed on a technical balance (Radwag, Radom, Poland). Mixing was conducted on a powder mixer (Pharmalabor, Canosa di Puglia, Italy) for 20 min, at maximum mixing speed (5/5, 130 rpm) and using a mixing container whose volume was approximately half filled with the powder. The composition of the solid-filled capsules is shown in Table 1.

Additionally, three liquid-filled and three semisolid-filled (with the addition of colloidal silicon dioxide) formulations were compounded.

Formulations with liquid filling (F_3_, F_4_ and F_5_) were compounded by measuring the ALA needed to compound 50 capsules (5 g) and dissolving in a required volume of absolute ethanol, PEG 400 or caprylic/caprylic triglycerides. The required volume corresponded to 70% of the capsule volume, i.e., 476 µL per capsule (23.8 mL for 50 capsule size 0). Mixing was performed on a magnetic stirrer (Boeco, Hamburg, Germany) at room temperature for 2 h. After mixing, clear yellow solutions were obtained with absolute ethanol and PEG 400, while mixing ALA with caprylic/caprylic triglycerides resulted in obtaining a suspension. Using an automatic pipette (Brand, Wertheim, Germany), 476 µL of the resulting solution or suspension was transferred to the open capsules on the manual capsule filling machine and the capsules were closed. Before sealing the capsules, silicon dioxide was added in semisolid-filled capsule formulations (F_6_, F_7_ and F_8_) to the full volume with shaking on a vibrating plate (Retsch, Hahn, Germany). The composition of the capsules with liquid and semisolid matrix are shown in Table 2.

### 2.3. Flowability

The flowability determination of ALA, CapsuLac^®^ 60, Excipient II, and the mixture for the preparation of formulations F_1_ and F_2_ was performed on a compaction apparatus (Jolting volumeter, stav II, J. Engelsmann AG, Ludwigshafen, Germany). A measuring cylinder of 10 mL was used, and the mass of the sample was 2 g. Bulk volume and tapped volume after 10, 100, 500, 900, and 1250 impacts were recorded. All measurements were performed in triplicate, and results are presented as mean ± standard deviation. The flowability of the powder was estimated based on the values of the Compressibility Index and the Hausner Ratio, which were calculated according to the guidelines given in the 11th European Pharmacopoeia (Ph. Eur. 11) [37], as shown in Equations (2) and (3):

(2) $$Compressibility\;Index=100\times \frac{V_{0}-V_{f}}{V_{0}},$$

(3) $$Hausner\;Ratio=\frac{V_{0}}{V_{f}}$$

where V_0_ is the unsettled apparent volume and V_f_ is the final tapped volume.

Additionally, for the same substances and mixtures, the angle of repose (α) was determined according to the recommendations given in Ph. Eur. 11. (Equation (4)). The test was performed in triplicate, and the results are presented as mean ± standard deviation [37].

(4) $$tan\left(\alpha \right)=\frac{height}{0.5\times base}$$

### 2.4. Uniformity of Mass

Uniformity of mass was conducted according to Ph. Eur. 11. Twenty randomly selected capsules were weighed on an analytical balance (Radwag, Radom, Poland) before and after careful emptying of the capsule filling. From the difference in the two values, the average value of twenty measurements was calculated. It was observed whether the individual masses deviated more than the permitted percentage deviation (7.5%, average masses greater than 300 mg) [37].

### 2.5. Assay

The filling of 10 individual capsules was carefully dissolved in 10 mL of methanol. The samples were exposed to ultrasonic waves for 15 min without heating (Bandelin Sonorex, Berlin, Germany). The content in the prepared samples was determined spectrophotometrically (G1103A, Agilent Technologies, Santa Clara, CA, USA) [38]. The absorbance of the solution was measured at 330 nm. The dependence of absorbance on ALA concentration was linear in the concentration range 0.078125–2.5 mg/mL (R^2^ = 0.99988). The content is expressed as a percentage of the declared (theoretical) value.

### 2.6. Fourier Transform Infrared Spectroscopy

The infrared spectra were obtained using a Fourier transform infrared spectrophotometer (FTIR) IRAffinity-1S (Shimadzu, Tokyo, Japan). Measurements were carried out using ATR (attenuated total reflection) in the range 4000–400 cm^−1^, with a resolution of 4 cm^−1^.

### 2.7. In Vitro Dissolution Study

The selection of the medium for testing the dissolution rate was based on the study of the solubility and stability of ALA. Solubility was tested in water, pH 1.2 and pH 6.8 mediums without and with addition of sodium lauryl sulfate (SLS) in the concentrations of 0.1, 0.25, and 0.5%. Solubility was tested in triplicate after 24 h in a thermostatic water bath with a shaker (WSB-18, Witeg Labortechnik, Wertheim, Germany) at a temperature of 37 °C. The stability of the solution was tested by re-measuring the content in the samples after an additional 24 h at a temperature of 37 °C. As a medium, a 0.5% aqueous solution of sodium lauryl sulfate (SLS) was chosen.

The dissolution rate test was performed using a basket apparatus on a dissolution rate test device (DT 800 LH, Erweka^®^, Heussenstam, Germany). The volume of the medium was 500 mL, at a temperature of 37 °C, and at a rotation speed of 100 rpm. Sampling was performed at 5, 15, 25, 35, 45, and 60 min from the start of the test. The samples were filtered prior to analysis through membrane filter size 0.45 µm (Sartorius Lab Instruments, Göttingen, Germany). The concentration of ALA at the observed time points was determined by the aforementioned spectrophotometric method.

### 2.8. Rheological Properties

HAAKE MARS rheometer (Thermo Scientific, Karlsruhe, Germany) was used for the investigation of rheological behavior. Measurements were performed 24 h after compounding at room temperature (25 ± 0.1 °C) using cylinder CC25 DIN/Ti for liquid samples and plate–plate PP35 Ti measuring geometry with a gap of 1.000 mm for semisolid samples. Continual increase in shear rate from 0.005 to 200 s^−1^ during 120 s was carried out with the purpose of conducting the hysteresis loop test. Afterwards, the shear stress was constant for 60 s at 200 s^−1^ and lastly, it decreased to 0 s^−1^ during the next 120 s.

Linear viscoelastic region (LVR) was determined by amplitude sweep test where storage (G′) and viscous modulus (G″) were recorded versus shear stress (0.01–10 Pa) at constant frequency of 1 Hz. Frequency sweep tests were performed based on the strain, selected as middle of LVR. G′ and G″ moduli were recorded versus frequency (0.1–10 Hz) at constant shear stress.

## 3. Results

### 3.1. Flowability

As expected, pure ALA has the worst flowability according to all three observed parameters. Interestingly, the combinations of ALA and selected diluents have a better flowability than pure diluents according to the Hausner Ratio and Compressibility Index. Excipient II has poor flowability compared to CapsuLac^®^ 60. Consequently, the formulations containing Excipient II (F_2_) are characterized by worse flowability compared to the formulation containing CapsuLac^®^ 60 (F_1_). The values of the Compressibility Index, Hausner Ratio, and angle of repose of pure substances and tested mixtures F_1_ and F_2_ are shown in Table 3.

### 3.2. Uniformity of Mass and Assay

Uniformity of mass as well as content uniformity are in accordance with the requirements of Ph. Eur. 11. The F_2_ formulation has a greater mass variation compared to F_1_ formulation, which may be related to the worse flowability. The results of uniformity of mass and assay of the prepared formulations are shown in Table 4.

### 3.3. Fourier Transform Infrared Spectroscopy

**Figure 2:**
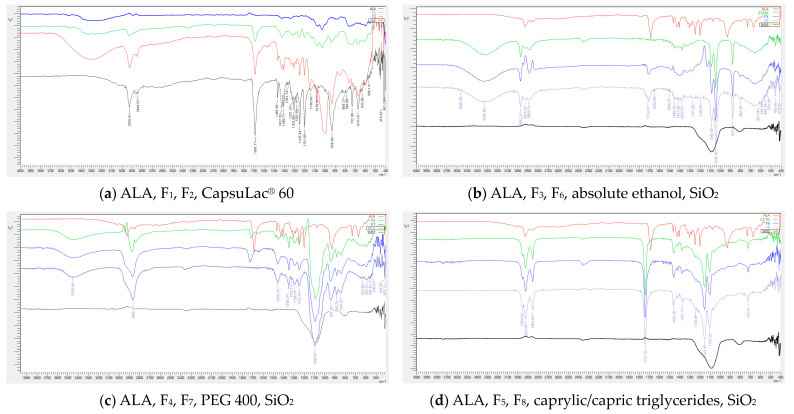
The FTIR spectra of (**a**) solid-filled capsules; (**b**) capsules with absolute ethanol; (**c**) capsules with PEG 400; and (**d**) capsules with caprylic/capric triglycerides.

The FTIR spectra of ALA and other ingredients with appropriate formulations are shown in Figure 2. The band in ALA spectrum at 2926 cm^−1^ corresponds to the methylene stretching and does not change after the ALA formulation. Also, bands at 2850 and 2920 cm^−1^ for symmetric and asymmetric ν(C–H) vibrations, respectively, are presented in pure ALA and all formulations. A prominent band at 1687 cm^−1^ is associated with the C=O stretching vibrations from the carbonyl group of ALA as shown in Figure 2. The OH stretching from the –COOH group presents at 1465, 1427, and 1247 cm^−1^ from ν(C–O)/δ(OH) out-of-plane, with strong absorption at 929 from δ(OH) out-of-plane. The position of these characteristic bands of –OH group in ALA did not change in the spectra of the F_1_ and F_2_ formulations as shown in Figure 2a. However, the S–S and C–S bands were observed at 673 cm^−1^ from ν(C–S) and at 499 and 451 cm^−1^ from ν(S–S) [39,40]. Figure 2a illustrates that there was no typical band appearance or disappearance of ALA in the F_1_ and F_2_ formulation, demonstrating the existence of ALA. In F_1_, peaks from lactose are presented at 3260 cm^−1^ from OH vibration, 1070 to 1030 cm^−1^ from C–O–C and C–OH groups of the lactose. Other lactose peaks overlap with intensive peaks of ALA. In the F_2_ formulation, characteristic peaks from corn starch are presented at 3250 cm^−1^ from O-H stretching, C–O–C asymmetric stretching at 1149 cm^−1^ and 1078 and 1014 cm^−1^ originated from C–O stretching. These peaks are presented in F_2_ without shifting.

In the F_3_ and F_6_ formulations, shifting of the characteristic ALA peaks is observed as follows: C=O carbonyl from 1687 to 1714 cm^−1^, as well as peaks from ν(C–O)/δ(OH) out-of-plane from 1465, 1427 and, 1247 cm^−1^ in pure ALA to 1456, 1417, and 1271 cm^−1^ in formulation, which confirmed the formation of a supramolecular structure with ethanol. The formation of a complex structure is also confirmed by the disappearance of the strong absorption at 929 cm^−1^ from δ(OH) out-of-plane in pure ALA. Other characteristic peaks of ethanol are present in the F_3_ and F_6_ formulation at 879 and 1045 cm^−1^ from C–C–O stretching, 1085 cm^−1^ from C–O stretching, CH_3_ rocking and at 3332 cm^−1^ from the OH group in ethanol.

In the F_4_ and F_7_ formulation, shifting of the C=O carbonyl from 1687 (in pure ALA) to 1728 cm^−1^ confirms the interaction of PEG and ALA. PEG characteristic peaks presented in the F_4_ and F_7_ formulation at 3456 cm^−1^ are ascribed to O–H stretching. The absorption at 1456 cm^–1^ was designated to the C–H bending vibrations of the –CH_2_ group; the peak at 941 cm^−1^ was associated with the –CH out-of-plane bending vibrations of PEG. The C–O–C ether stretching absorption band at 1095 cm^−1^ overlapped with peaks from ALA ν(C–O)/δ(OH) out-of-plane (at 1465, 1427 and 929 cm^−1^).

The formation of a supramolecular structure of ALA with caprylic/capric triglycerides filler is confirmed by FTIR spectra, as shown in Figure 2d. In the F_5_ and F_8_ spectra, characteristic peaks from ALA disappear and the formulation spectra are the same as caprylic/capric triglycerides filler. A characteristic peak at 1741 cm^−1^ from carbonyl C=O, as well as peaks at 1103 and 153 cm^−1^, originating from C–O–C ester group vibration, are present in the spectra of pure caprylic/capric triglycerides and both formulations F_5_ and F_8_.

The peaks from SiO_2_ are not noticeable in any spectrum of the obtained formulations, as can be clearly seen in Figure 2. This is due to the absence of the peak at 1085 cm^−1^ originating from the Si–O–Si asymmetric stretching vibration and 804 cm^−1^ from the in-plane bending vibrations of geminal groups.

### 3.4. In Vitro Dissolution Study

**Figure 3:**
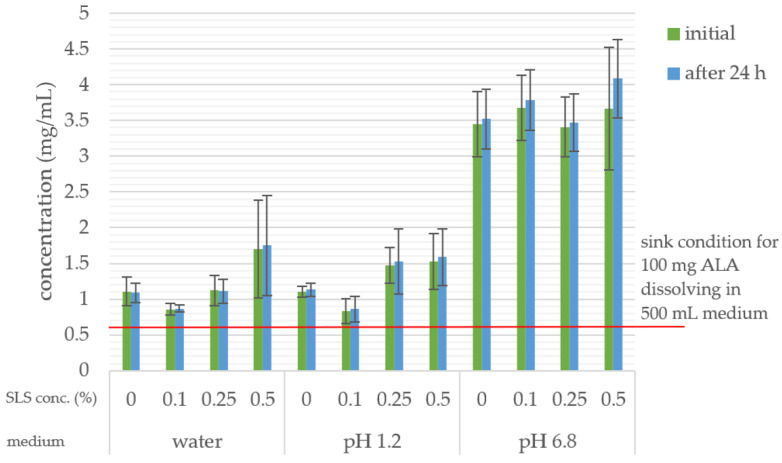
The solubility of ALA after 24 h in tested media (initial), and the results of repeated measurement of concentrations after an additional 24 h in order to test stability.

The solubility of ALA in all tested media (distilled water, pH 1.2 media, and pH 6.8 phosphate buffer) with and without SLS was sufficient to provide sink conditions for the dissolution of 100 mg ALA in 500 mL of media (the solubility is higher than the concentration obtained by dissolving the triple dose in the medium). The concentration of ALA was unchanged after 24 h of storage at 37 °C (*p* < 0.05). The solubility results are shown in Figure 3.

**Figure 4:**
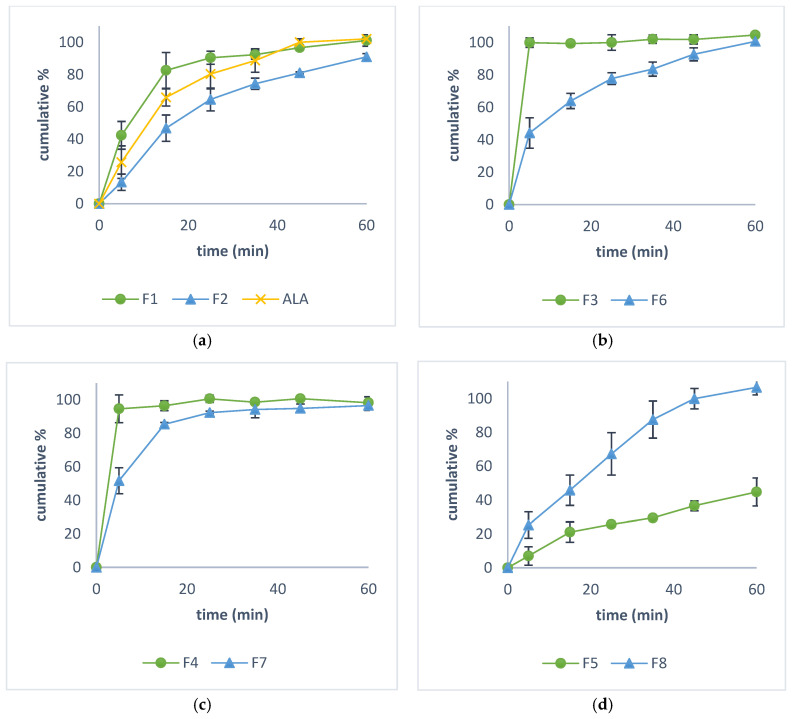
The dissolution rate profiles of (**a**) solid-filled capsules; (**b**) capsules with absolute ethanol; (**c**) capsules with PEG 400; and (**d**) capsules with caprylic/capric triglycerides.

Capsules with pure ALA have a similar dissolution rate compared to ones with CapsuLac^®^ 60 as a diluent. However, using Excipient II as a diluent slows the release of ALA from the investigation solid-filled capsules, as shown in Figure 4a.

The release of ALA from the formulation filled with solutions (F_3_ and F_4_) is practically instantaneous and after 5 min the complete release of the contents is achieved. As expected, the addition of silicon dioxide to the formulations results in slower release due to increased viscosity. The mentioned effect is more pronounced in the formulation with ethanol (F_6_), where 63.86% of the content was released after 15 min, than in the formulation with PEG 400, where 85.37% of the content was released in the same time. The dissolution rate of ALA from the formulation filled with suspension (F_5_) was the slowest (only 44.77% after 60 min). Interestingly, the addition of silicon dioxide to formulation F_5_ (F_8_) led to an improvement of ALA release rate (complete content after 60 min was dissolved), contrary to the previous two formulations with liquid contents. The dissolution rates curves are presented in Figure 4.

### 3.5. Rheological Properties

**Figure 5:**
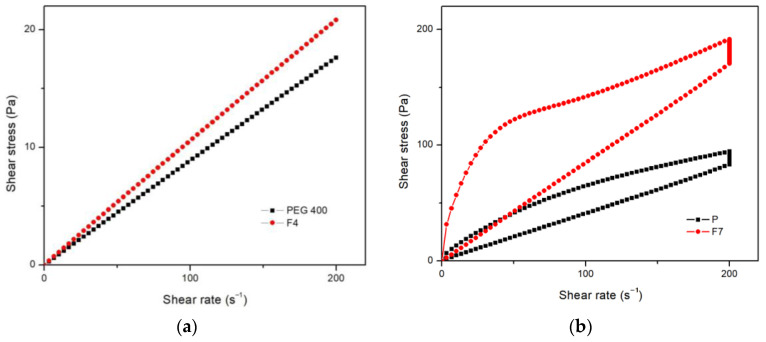
Rheograms of shear stress versus shear rate of (**a**) ALA solution in PEG 400 (F_4_) and pure PEG 400; (**b**) ALA silicon dioxide gel formulation in PEG 400 (F_7_) and corresponding placebo formulation without ALA (P).

The linear dependence of shear stress on shear rate in pure PEG 400 and ALA solution in PEG 400 indicates their Newtonian behavior. The R-squared value of 1 was attained when experimentally determined curves were fitted via the equation of Newton’s law. The measured viscosity of PEG 400 is 87.6 ± 0.6 mPa·s. The character of the flow behavior was not changed after the addition of ALA, but the viscosity was increased to 102.9 ± 1.7 mPa·s as presented in Figure 5a.

The addition of silicon dioxide to PEG 400 led to a change in both flow character and viscosity. Non-Newtonian, time-dependent, thixotropic flow behavior was observed in the placebo gel formulation (P). The same as in pure PEG 400, the addition of ALA (F_7_) to the placebo gel formulation increased the viscosity without changing the flow type, as shown in Figure 5b. Thixotropy is more pronounced in formulation F_7_, considering the area of the hysteresis loop. The area of the hysteresis loop is significantly larger in formulation F_7_ (10430 ± 151 Pa·s), compared to the placebo gel formulation P (3701 ± 11.79 Pa·s).

**Figure 6:**
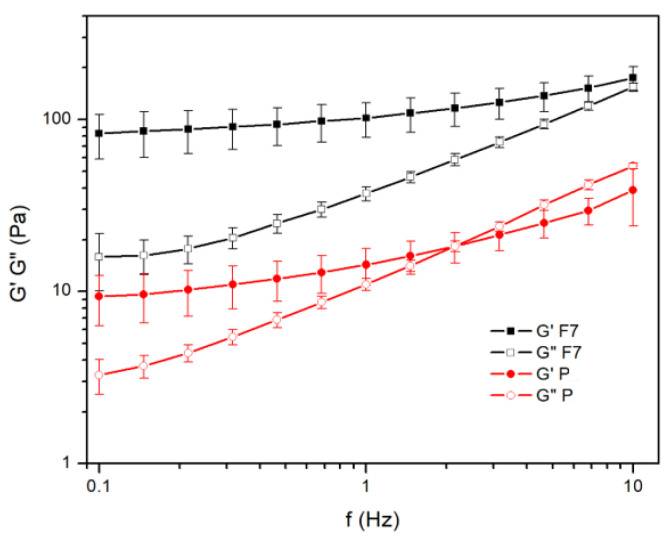
Effect of ALA on changes in G′ and G″ modulus versus frequency.

In the placebo gel formulation, which consisted only of PEG 400 and silicon dioxide (P), G′ dominates over G″ at low frequencies. The cross-over at about 2.15 Hz occurs and after that, G” continues to be dominant. In the semisolid formulation with ALA (F_7_), G′ is more pronounced compared to G″ for the entire observed range of frequencies (Figure 6).

G″ to G′ ratios (tan δ) are from 0.19 ± 0.02 to 0.90 ± 0.11 for F_7_ formulation and from 0.36 ± 0.07 to 1.49 ± 0.57 for placebo (P) formulation without ALA indicating their weak gel structures.

## 4. Discussion

ALA is a specific compound as it has authorized formulations of drugs as well as food supplements and it is used as a potential therapeutic agent for many chronic diseases with potential epidemiological, economic and social impact. Authorized formulations contain multiple excipients and usually dose above 200 mg. Taking into account the prolonged use of ALA formulations and its pleiotropic effect, there is a need for the compounding of simplified formulations. As indicated before, ALA is a poorly soluble molecule; thus, in the literature, there are various techniques used to increase ALA solubility, but they have some drawbacks and disadvantages. Many solubility enhancement techniques are costly processes that are often difficult to carry out and have poor manufacturability. In this research, simple formulations were prepared, suitable for compounding.

Powder formulations in this study were prepared by mixing ALA with CapsuLac^®^ 60 or Excipient II as fillers. As expected, the type of filler influenced flowability and dissolution properties of ALA. An interesting finding was that the addition of ALA to solid fillers (i.e., CapsuLac^®^ 60 and Excipient II) improved their flowability. Though, ALA has very poor flowability (Hausner Ratio (1.51) and Compressibility Index (33.63%), Table 3) and when added to diluents, it impacts their flowability. Another interesting result is that ALA increases the flowability of both diluents, though their chemical composition and particle size and shape is different. Flowability is a critical quality attribute in hard capsules formulations, and this effect of ALA is seen as positive, thus there is no need for the determination of the percolation threshold [41]. As expected, since ALA did not have a negative impact on flowability, both formulations ensure the adequacy of the uniformity of mass and the assay tests. However, smaller variations were observed in formulation F_1_, which had better flowability. The individual masses of the capsules varied in the range of 98.42–102.03% in the F_1_ formulation, while in the F_2_ formulation they were in the range of 95.37–102.21% in relation to the average mass. Similar results were obtained in the assay test, where smaller standard deviations were observed in formulation F_1_ (1.38%) than in formulation F_2_ (2.05%). The suitability of using CapsuLac^®^ 60 and Excipient II as fillers in capsule compounding has been previously confirmed. CapsuLac^®^ 60 and Excipient II have proven to be excipients that provide adequate flowability and compliance with pharmacopeial requirements in terms of mass and content variation, as in the example of pantoprazole low-dose capsules compounding [42].

The dissolution rate of formulations where CapsuLac^®^ 60 was used as filler was faster than those with Excipent II. This was also noticed in our previous study [42]. Also, it is notable that, compared to pure ALA, mixture with Excipient II decreased the dissolution rate, but mixture with CapsuLac^®^ 60 increased the dissolution rate. The reason for this might be the composition of fillers. Namely, Excipient II contains pregelatinized corn starch and micronized silicon dioxide, which first forms a gel structure in dissolution medium and then dissolves. FTIR spectra did not detect any interaction with the investigated fillers. The shells were not expected to interact with the content. Also, the rapid and complete disintegration and dissolution of gelatin shells in tested conditions has been well documented in previously published studies [43,44].

Additionally, though ALA’s lack of gastric stability has been implied in other studies [30,45], in our study ALA was stable in pH 1,2 medium at 37 °C with and without SLS (0.1%, 0.25%, and 0.5%) for 24 h. Also, ALA was stable at 37 °C for 24 h in water and pH 6.8 buffer, with and without SLS.

Liquid formulations were prepared because, according to the literature, bioavailability could be markedly increased when ALA is orally administered in the liquid form rather than a solid dosage form. Higher plasma concentrations and accelerated absorption of ALA is expected from liquid formulations [30,45]. Both types of vehiculum and whether ALA is dissolved or suspended have an impact on dissolution rate. Formulations with absolute ethanol (F_3_) and PEG 400 (F_4_) released the entire ALA content in the first 5 min. Formulations where ALA was suspended (i.e., with caprylic/caprylic triglycerides, F_5_) showed significant retardation in dissolution rate, compared to formulations F_3_ and F_4_ where ALA is dissolved. F_5_ released only 7% of ALA content after 5 min. The lipophilicity of the medium and slower mixing with the aqueous dissolution medium, as well as the lower affinity of ALA to migrate can be the reasons for the slow release obtained. Additionally, the surface area available for dissolution is smaller in suspension formulations, and the particle size is one of the key factors affecting the dissolution process [46].

An increase in viscosity was observed in all formulations where silicon dioxide was added (F_6_, F_7_ and F_8_), but the effects on dissolution rate were not equal. Namely, an increase in viscosity was expected to be inversely proportional to the dissolution rate [47]. When silicon dioxide is added to formulations in which ALA is dissolved, the aforementioned deceleration of release occurs, as shown in Figure 2a,b. However, after adding silicon dioxide to the formulation with PEG 400, 85.37% of the ALA content was released after 5 min. Although a decrease in the dissolution rate of ALA is observed, it is not statistically significant considering the guidelines given by the European Medicines Agency (EMA). According to these guidelines, for fast-release formulations where more than 85% of the content is released within 15 min, no mathematical considerations are needed and their release profiles can be considered similar [48]. Consequently, it can be concluded that the observed increase in viscosity in the formulation with PEG 400 (F_4_) after the addition of silicon dioxide (F_7_) did not lead to a statistically significant decrease in the dissolution rate of ALA. Interestingly, the release profile improved when silicon dioxide was added to the formulation in which ALA was suspended. The reason for this may be the stabilization of the suspension, which prevents the agglomeration of ALA particles, as well as increased wetting. It could be concluded that PEG 400 was most suitable as the vehicle for liquid compounding.

Based on the results of previously published studies [49,50,51,52], the viscosity of ALA solution in PEG 400 is suitable for filling hard gelatin capsules. The moderate viscosity of the solution of ALA in PEG 400 is desirable due to the avoidance of loss caused by splashing during the filling process. Also, the addition of silicon dioxide after and not before filling capsules provided a viscosity increase after precise dose filling and problems that were observed with high viscosity filling materials was avoided. Silicon dioxide contains colloidal, nanometer-sized primary particles with superficial silanol groups, which makes it possible to build hydrogen bonds with PEG 400 and form cross-linked gel networks [53]. The addition of ALA in the system with silicon dioxide and PEG 400 showed G’ dominating over G” in the entire tested frequency range, without the characteristic cross-over observed with the placebo (P) formulation. A potential reason why the elastic part dominates over the viscous part in formulation F_7_ could be the high concentration of ALA and its contribution to the increase in viscosity (Figure 5a). The presence of ALA in the formulation contributes to the increase in elastic (gel) properties. However, the ratio of G″ to G′ (tan δ) are higher than 0.1 and the F_7_ formulation cannot be considered as a true rather than as a weak gel [54,55,56]. The obtained rheological behavior can be considered as a desirable one, because it did not statistically significantly affect the dissolution rate of ALA. Based on all the above, it is concluded that the applied capsule filling technique is suitable for compounding.

## 5. Conclusions

This research aimed to provide a thorough and complete characterization of compounded hard gelatin capsules containing ALA dose of 100 mg. Capsules with liquid and semisolid content were proven to be suitable for compounding as ones with conventional solid content filing. All formulations complied to pharmacopeial requirements. However, dissolution profiles were faster in formulations filled with liquid content compared to solid-filled formulations. Although the addition of silicon dioxide led to an increase in viscosity and a change in rheological behavior, the release rate did not decrease significantly. PEG 400 (formulation F_4_) proved to be the most adequate liquid medium for compounding since ALA was completely dissolved in it and FTIR analysis did not indicate interactions. Subsequently adding silicon dioxide (formulation F_7_) in order to form semisolid content is adequate for preventing content leaking without impacting the filling process.

## Figures and Tables

**Table 1:** Composition of the solid-filled capsules.

Formulation	Ingredient		
	ALA	CapsuLac^®^ 60	Excipient II
F_0_	100 mg	/	/
F_1_	100 mg	q.s. *	/
F_2_	100 mg	/	q.s.

* q.s.—quantum satis.

**Table 2:** Composition of the liquid- and semisolid-filled capsules.

Formulation	Ingredient				
	ALA	Absolute Ethanol	PEG 400	Caprylic/Capric Triglycerides	Silicon Dioxide
F_3_	100 mg	ad 476 µL	/	/	/
F_4_	100 mg	/	ad 476 µL	/	/
F_5_	100 mg	/	/	ad 476 µL	/
F_6_	100 mg	ad 476 µL	/	/	q.s.*
F_7_	100 mg	/	ad 476 µL	/	q.s.
F_8_	100 mg	/	/	ad 476 µL	q.s.

* q.s.—quantum satis.

**Table 3:** Character of flowability of tested powdery substances and mixtures.

Substance/ Mixture	Hausner Ratio		Compressibility Index (%)		Flow Character According to Hausner Ratio and Compressibility Index	Angle of Repose		Flow Property According to Angle of Repose
	Mean	SD	Mean	SD		Mean	SD	
ALA	1.51	0.01	33.63	0.30	Very poor	49.09	0.55	Poor
CapsuLac^®^ 60	1.31	0.02	23.80	1.21	Passable	32.87	1.43	Good
Excipient II	1.43	0.04	30.26	2.11	Poor	36.71	0.79	Fair
F_1_	1.14	0.03	12.08	2.22	Good	39.74	0.29	Fair
F_2_	1.29	0.01	22.43	0.37	Passable	41.22	0.36	Passable

**Table 4:** Results of uniformity of mass and assay test.

Formulation	Uniformity of Mass (%)		Assay (%)	
	Min	Max	Mean	SD
F_1_	98.42	102.03	96.61	1.38
F_2_	95.37	102.21	97.79	2.05
F_3_	98.33	105.98	98.93	0.97
F_4_	95.72	106.11	98.09	2.45
F_5_	95.19	107.27	98.17	1.09
F_6_	97.43	104.68	96.05	0.84
F_7_	95.63	104.95	101.18	3.01
F_8_	96.44	104.99	99.82	2.17

## Data Availability

The original contributions presented in the study are included in the article, further inquiries can be directed to the corresponding author.
